# Systems vaccinology analysis of saRNA immunization identifies an acute innate immune signature correlated with adaptive immunity

**DOI:** 10.1016/j.omta.2026.201706

**Published:** 2026-02-20

**Authors:** Tamara Elliott, Ziyin Wang, Olivia Bonduelle, Abbey Evans, Suzanne Day, Leon R. McFarlane, Simon de Bernard, Karine Alves, Julien Nourikyan, Michele Wokam, Katrina Pollock, Hannah M. Cheeseman, Behazine Combadiere, Robin J. Shattock, John S. Tregoning

**Affiliations:** 1 Department of Infectious Disease, Imperial, London, UK; 2 Sorbonne Université, Institut National de Santé et de Recherche Médicale - Inserm U1135, Centre d’Immunologie et des Maladies Infectieuses - Cimi-Paris, 75013 Paris, France; 3 Altrabio, 69007 Lyon, France

**Keywords:** systems vaccinology, RNA vaccine platform, transcriptomic, SARS-CoV-2, self-amplifying RNA, early innate immune activation, host response profiling, ISG

## Abstract

Self-amplifying ribonucleic acid (saRNA) vaccines are a next-generation RNA vaccine platform with great potential. Systems vaccinology provides a potent tool to interrogate vaccine-induced responses in volunteers and to dissect the mechanisms by which vaccines elicit a protective immune response or cause reactogenicity. In the current study, we performed transcriptomic analysis on blood samples collected from volunteers vaccinated as part of a phase I study of an saRNA vaccine expressing the severe acute respiratory syndrome coronavirus 2 (SARS-CoV-2) spike antigen. We observed significant gene over-expression following both the prime and boost vaccinations. Over-expressed genes were predominantly associated with type I interferon signaling pathways and innate immune cell recruitment. This transcriptomic signature was reflected by an increase in cytokines in the plasma at the same time points and a significant increase in monocytes in the blood, both of which correlated with the antibody response to the vaccine. When individuals were segregated by the degree of reactogenicity, we also detected differences in gene expression related to immune responses. Overall, results show that saRNA induces a potent, acute inflammatory response with similarities to other RNA vaccines, and it will be important to further dissect the role of the over-expressed genes in immunogenicity and reactogenicity.

## Graphical abstract

**Figure:**
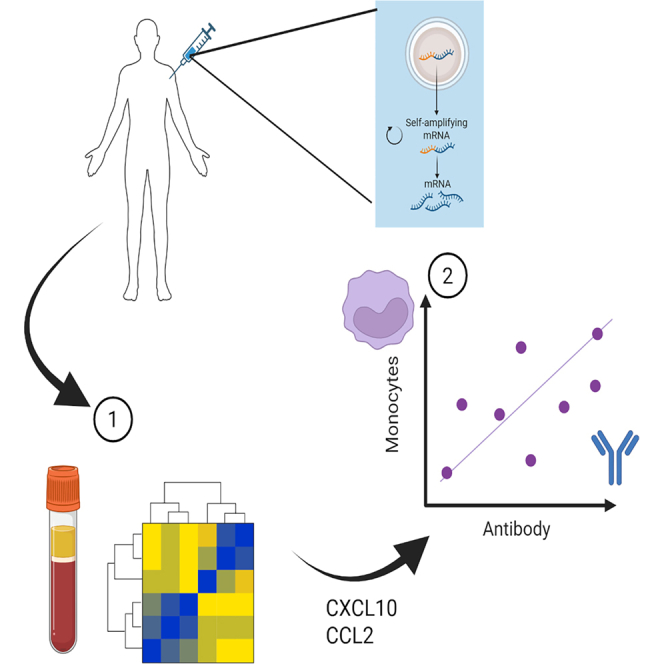

## Teaser

Self-amplifying RNA vaccines offer considerable benefits through dose reduction. Acute host responses to RNA vaccination shape downstream adaptive immune responses. In this study, Elliott et al. measured acute changes in transcriptomics during an saRNA clinical trial and showed a strong interferon signature that correlated with the peak antibody response.

## Introduction

The infection threats faced—HIV, TB, malaria, emerging pandemic viruses, and antimicrobial-resistant bacteria—demand new vaccine approaches. Coronavirus disease 2019 (COVID-19) has highlighted the potential of RNA vaccination as a flexible and rapid platform to control emerging infections. The first experimental severe acute respiratory syndrome coronavirus 2 (SARS-CoV-2) mRNA vaccine was administered 63 days after the publication of the viral sequence.^1^ However, RNA vaccines are still a relatively new platform, and further research is needed to deliver protection against a broader range of pathogens. An extremely promising alternative to mRNA vaccines is self-amplifying ribonucleic acid (saRNA), a platform derived from alphavirus genomes that utilizes a replicase complex to amplify the vaccine antigen. A major benefit of saRNA is dose reduction. We have seen this dose reduction in pre-clinical models, where equivalent immunogenicity was induced with 100-fold less saRNA than mRNA,^2^ and protection was achieved with as little as 0.01 μg of saRNA.^3^ Reduced dose translates to more vaccine per production run, accelerating rollout in a pandemic.

However, further optimization is required for the saRNA platform. Moving from mouse models into human clinical trials revealed some limitations with the first iterations of saRNA vaccines. When used in an antigen-naive population, an saRNA vaccine encoding SARS-CoV-2 spike did not induce an immune response in all volunteers. Only 87% of vaccinated individuals seroconverted in the initial phase I clinical trial^4^ and 80% in a follow-up phase 2a study.^5^ A similar 80% seroconversion rate was seen for a different replicating RNA vaccine expressing spike.^6^

RNA vaccines differ from earlier-generation vaccines because they need to be translated *in situ* before the antigen is detected by the adaptive immune system. If the RNA is not translated and the antigen is not produced, there can be no adaptive immune response. One aspect of the response to RNA vaccines that may particularly impact antigen expression is the type I interferon (IFN) response, which induces an anti-viral state, inhibiting a range of cellular functions that viruses otherwise hijack to replicate, including protein translation. Cell models can provide some insight into the interplay of IFN and gene expression from saRNA vaccines.^7^ However, antigen expression is only half of the RNA vaccine story. Antigen that is produced but not detected by the immune response is no more effective than antigen that is never produced.^8^ The recruitment of innate immune cells to the site of immunization is vital. One way to interrogate the human immune response is through blood transcriptomics, which allows a much more detailed investigation of the acute immune response to immunization.^9^ Blood transcriptomics has been applied to several platforms, including RNA vaccines, providing insight into the response. In the current study, we examined a subset of volunteers from the COVAC1 saRNA vaccination trial (ISRCTN17072692, EudraCT 2020-001646-20), in which volunteers received two doses of an saRNA vaccine intramuscularly. Samples were collected at prime and boost for RNA sequencing (RNA-seq) and flow cytometry analysis, as well as for antibody and T cell responses to the vaccine. We observed a robust transcriptomic response, associated with an increase in innate immune cells in the blood 24 h after vaccination, which correlated with the antibody response.

## Results

### Experimental setup

**Figure 1:**
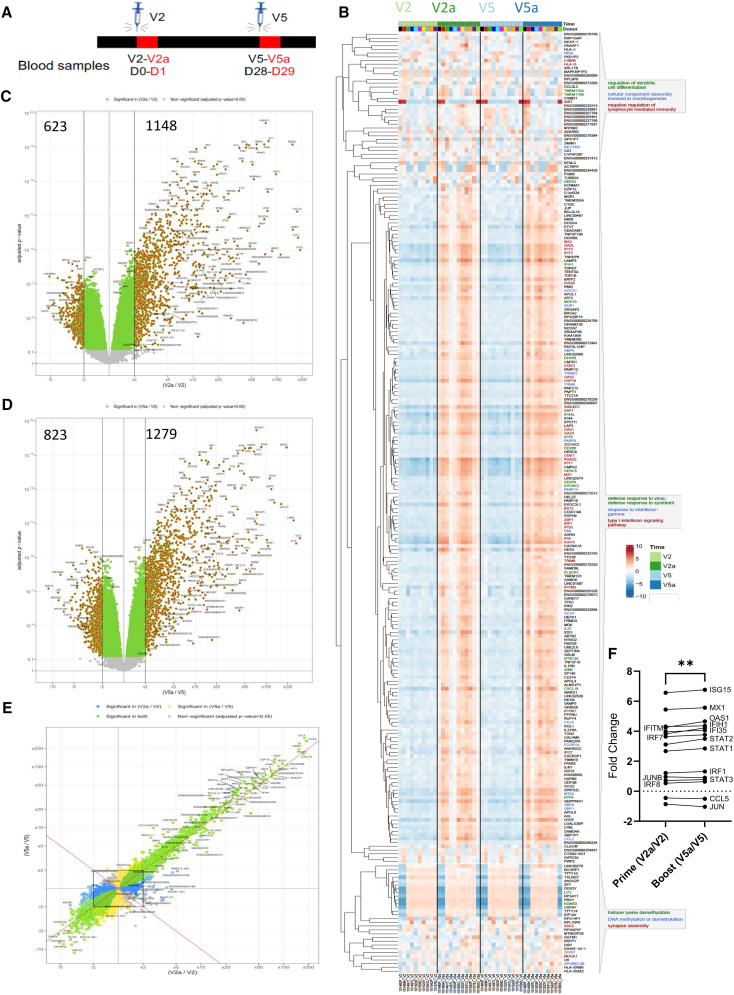
saRNA immunization leads to significant gene expression 24 h after prime and boost (A) Blood sampling schedule. (B) Heatmap of the genes with the highest variance in the experiment, associated with type 1 IFN. (C) Volcano plot of DEG at prime immunization; 1,769 DEG. (D) Volcano plot of DEG at boost immunization; 2,009 DEG. (E) Comparison of pre-and post-injection effects at prime and boost. Spot legend: Yellow – significant on the *y* axis (boost only); Blue – significant on the *x* axis (prime only); Green – significant on both. (F) Change in interferon response gene signature based on Arunachalam et al. (Points represent mean DEG fold change of each gene, ∗∗*p* < 0.01).

The aim of the study was to investigate changes in gene expression following immunization with an lipid nanoparticle (LNP)-formulated saRNA expressing the SARS-CoV-2 spike antigen. Samples were collected from 11 individuals who were unexposed to the SARS-CoV-2 virus and had not received any other vaccine. Volunteers were immunized in a prime-boost regime with either 1 or 5 μg of saRNA, with variable intervals between vaccinations (Table S1). Blood was collected into PAXgene tubes for later RNA extraction on the day of and 24 h after both immunizations (Figure 1A); sampling time points are part of a larger schedule and referred to as V2 (time of prime), V2a (prime plus 24 h), V5 (time of boost), and V5a (boost plus 24 h). All RNA samples collected, except one, were of sufficient quality for sequencing. No significant contamination was observed in any of the samples. Samples for adaptive immune response assessment were collected at various time points after immunization. Participant numbers were limited by the wider rollout of licensed COVID-19 vaccines.

### saRNA immunization induces a greater response at boost than prime

Collected samples were analyzed by RNA-seq, which indicated that saRNA immunization causes significant changes in the blood transcriptome both after the prime and boost, and that these changes are broadly similar (Figure 1B). A heatmap was generated for the top 250 genes with the highest variance using Ward’s agglomerative method (Figure 1B). When grouped by sample time point, a clear pattern of over-expression in genes associated with the type I IFN signaling pathway was observed 24 h after either the prime or boost vaccination (V2a and V5a). Comparing the two baseline samples (V2 and V5), there were no genes that were significantly different. However, immunization led to a clear difference in gene expression at the 24-h time point. At the first (V2a) immunization, there were 1,148 over-expressed (adj *p* < 0.05, fold change >2) and 623 under-expressed (adj *p* < 0.05, fold change <-2) differentially expressed genes (DEGs) compared to the pre-immunization (V2) bleed (Figure 1C). The top genes by fold change were chemokines *CCL8*, *CCL2*, and *CXCL10* (Table 1). When split by dose received, the group that received the 5 μg dose had more DEGs than the group that received 1 μg (Figure S1A).

**Table 1:** Top 10 DEG by fold change, V2a to V2 (prime) and V5a to V5 (boost)

Prime				Boost		
Rank	Fold change	adj. p	Symbol	Fold change	adj. p	Symbol
1	189	1.07E−10	*CCL8*	296	2.2E−12	*CCL2*
2	179	8.74E−12	*CCL2*	263	6.1E−12	*CCL8*
3	174	4.59E−14	*CXCL10*	241	5.6E−14	*LAMP3*
4	155	1.77E−12	*LAMP3*	193	2.1E−20	*RSAD2*
5	153	5.86E−20	*RSAD2*	161	2.0E−15	*CXCL10*
6	111	2.70E−10	*LINC02068*	130	1.6E−15	*OTOF*
7	97	2.37E−17	*ISG15*	118	9.9E−17	*USP18*
8	88	1.28E−10	*HESX1*	112	1.0E−11	*ISG15*
9	79	2.95E−20	*IFIT1*	106	1.0E−11	*LINC02068*
10	78	8.41E−16	*USP18*	102	3.0E−12	*HESX1*

At the boost immunization, there were 1,279 over-expressed and 823 under-expressed DEGs (Figure 1D). The top 10 genes by fold change were very similar to those observed after the prime (Table 1). There were considerable overlap between the two immunizations, with 384 DEGs common to both; most overlapping DEGs shared the direction of fold change (Figure 1E). Notably, the magnitude of fold change was greater at boost than at prime, as has been observed in previous studies.^10^^,^^11^ We explored a subset of IFN response genes that Arunachalam et al.^11^ reported to be over-expressed following booster immunization and observed a modest increase in fold change at boost compared to prime (Figure 1F).

### saRNA immunization leads to cytokine gene upregulation and innate immune cell recruitment

Having observed significant changes in gene expression induced by saRNA vaccination, we investigated the function of the expressed genes. The MRGSE (Mean-Rank Gene Set Enrichment) approach was used to identify gene sets that were enriched. The top gene sets, using the Gene Ontology (GO) term assignment, were predominantly associated with immune response and signaling, including “innate immune response” (go:0045087), “cytokine-mediated signaling pathway” (go:0019221), and “response to IFN-gamma (IFN-γ)” (go:0034341). This indicates a role for genes associated with PAMP sensing and individual cytokine genes. To validate the RNA findings, plasma from volunteers at V2 and V2a was analyzed for cytokines using Meso Scale Discovery (MSD). There was a significant increase in several cytokines when comparing the supernatants between V2 and V2a (prime; Figure S1B) and V5 and V5a boost (Figure S1C). Both *CCL2* and *CXCL10* were significantly upregulated at the RNA and proteins levels, as was IFN-α. There was a trend toward higher levels of CCL2 and CXCL10 protein in the group receiving the higher dose (Figure S1D).

Having observed increases in cytokine gene transcription and significant changes in cytokine levels following stimulation of peripheral blood mononuclear cells (PBMCs) with saRNA, we explored whether there were changes in the cell profile in the blood after immunization. Frozen PBMCs collected at the V2, V2a, V5, and V5a time points were evaluated by spectral flow cytometry. Because this was 24 h after immunization, we focused only on innate cells. The supervised uniform manifold approximation and projection (UMAP) was used to analyze a subsample of 15,000 cells (excluding B and T cells) from all samples at each visit; this generated 12 main innate cell populations (Figure S2A). We then performed statistical analysis to determine whether there were significant changes in any of these 12 populations (Figure S2B). There was a trend toward a reduction in cDC1 and cDC2, significant increases in CD64^+^CD14^−^CD16^−^ monocytes at both vaccination time points, and an increase in pDC at boost.

### The relationship between adaptive response to vaccination and systemic inflammation

We evaluated the adaptive response to the encoded SARS-CoVC-2 spike antigen at various time points after vaccination, measuring both antigen-specific antibody and T cell responses. Due to the small number of participants, initial analyses were performed on pooled data. Because dosing regimens differed across individuals, results are presented relative to the vaccination visit.

**Figure 2:**
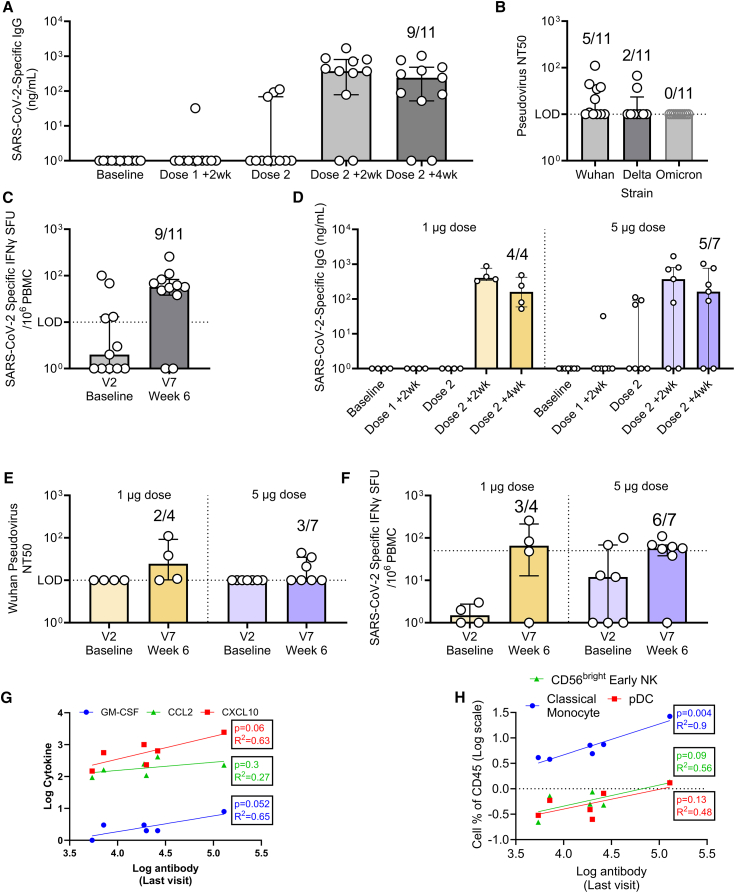
Adaptive immune responses to vaccination correlate weakly with innate responses 24 h after prime immunization Adaptive immune response data are presented from all participants pooled (A–C) or from participants separated into those who received 1 or 5 μg doses (D–F). (A and D) Anti-Spike (S) IgG (ng/mL) in sera from participants receiving two doses of LNP-saRNA at various time points after enrollment. (B and E) Pseudoneutralizing antibody IC50 from participants receiving two doses of LNP-saRNA; responses are shown at 6 weeks after enrollment against Wuhan, Delta, and Omicron spike-expressing pseudoviruses. (C and F) IFN-γ spot forming units (SFU) per million cells (ELISpot) from PBMC stimulated with SARS-CoV-2 spike peptide pools. (G) Correlation between V2a chemokine levels and the last visit antibody response. (H) Correlation between V2a cell levels and the last visit antibody response. Points represent individual participants, bars represent median ± interquartile range (A–F); points represent individual participants.

Anti-spike IgG was measured at various times after the prime and boost immunizations. No participant displayed a detectable IgG response after the first dose. Following the second dose, spike-specific IgG was detectable in 9 of 11 participants at both 2 and 4 weeks (Figure 2A). We also assessed functional antibody response in a pseudovirus assay. 5/11 participants had a measurable response against the original Wuhan viral variant, 2/11 against Delta, and 0/11 against Omicron (Figure 2B). T cell responses, measured using an IFN-γ ELISPOT, were detectable in 9/11 participants (Figure 2C). A few individuals displayed pre-existing T cell reactivity prior to vaccination, and overall, there was no correlation between antibody levels and IFN-γ T cell responses.

We next stratified the groups by the dose received to determine whether this had a substantial impact on the adaptive immune responses. The same kinetics of spike-specific IgG were observed in both groups, with responses after the second, but not the first, dose (Figure 2D). 100% (4/4) of the 1 μg group sero-converted following vaccination, while 71% (5/7) of the 5 μg group sero-converted. There was no significant difference in the titer, the number of individuals seroconverting, the neutralization titer (Figure 2E), or the participants with a T cell response (Figure 2F) when comparing individuals who received 1 or 5 μg dose. We then explored whether inflammation 24 h after immunization was associated with the downstream adaptive response. However, there was a correlation between V2a protein levels of GM-CSF and CXCL10 at V2a and the antibody response at the last visit (Figure 2G), with a weaker association observed for CCL2. When examining fold changes in *CCL2*, *CCL8*, and *CXCL10* transcripts relative to antibody titers, no direct correlations were observed. We also compared the cellular response at V2a with antibody responses. There was a correlation between CD56^bright^ NK cells and pDCs and the antibody response and a significant correlation between classical monocytes and antibody (Figure 2H).

Taken together—and acknowledging the limitation of the small sample size—we observed that levels of chemokines in the blood are associated with the antibody response to saRNA vaccination, with the caveat of the small sample size.

### Relation between vaccine-induced reactogenicity and transcriptomic changes at 24 h

**Figure 3:**
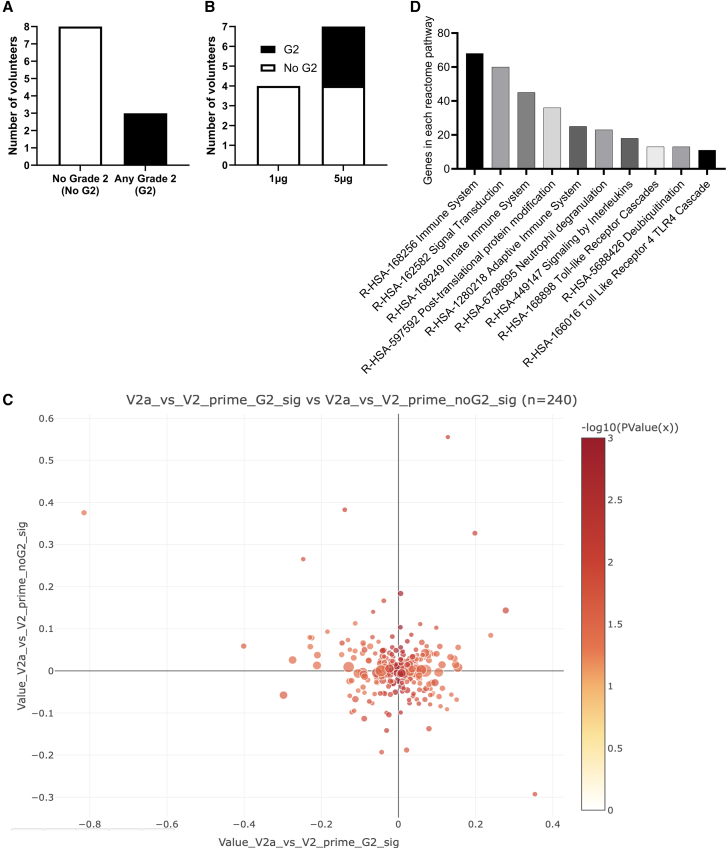
Reactogenicity is associated with immune-related gene upregulation (A and B) Individuals in the study were grouped by whether they had any adverse effects of grade 2 or greater after the first immunization (A); adverse effects by dose received (B). (C) DISCO analysis of DEG between V2 and V2a, comparing individuals with G2 adverse events with those without. (D) Reactome grouping of DEG unique to the G2 group. A,B bars represent numbers of participants, D bar represents number of genes in reactome pathway.

We wanted to assess whether there was a link between the adverse effects of vaccination and the transcriptomic signature. Overall, reactogenicity was mild; there were no grade 3 or grade 4 events. We grouped individuals by whether or not they had experienced any grade 2 adverse events, either systemic or local, after the first immunization (Figure 3A); 8 had not (no G2) and 3 had (G2). These were split by dose; all of the G2 individuals were in the 5 μg group (Figure 3B). In both groups, there were significant numbers of upregulated genes following immunization (Figures S3A and S3B); these separated between before and after immunization (Figures S3C and S3D), and the major determinants of PC1 were the same in both groups (Figures S3E and S3F; *ISG15*, *CXCL10*, *RSAD2*). We compared the DEGs between baseline (V2) and 24 h after immunization (V2a) in the G2 group and the no G2 group. There were a number of discordant genes (up in G2, down in no G2; Figure 3C). We then compared the over-expressed genes in the G2 group with those in the no G2 group; there were 179 shared DEGs, 322 unique to the no G2 group and 476 unique to the G2 group. Performing a Reactome clustering of the genes unique to the G2 group (Figure 3D), 68 genes clustered in the Immune System cluster, including *TLR3*, *TLR4*, *CCR2*, *RNASEL*, and *TBK1*. There were also clusters for signal transduction, adaptive immune system, and neutrophil degranulation (individual genes listed in Table S5). We also investigated whether this was reflected in the cytokines detected (Figure S4A), but there were no significant differences; neither were there differences in the proportions of cells in blood at V2a (Figure S4B).

## Discussion

In the current study, we explored acute blood transcriptomic changes after saRNA vaccination in a subset of volunteers from the COVAC1 phase 1 saRNA vaccine clinical trial. We observed significant changes in gene expression 24 h after either the prime or boost immunization. The majority of induced genes fell into immune response families, and this was reflected by an increase in cytokines from stimulated PBMCs and significant increases in innate cell numbers in the blood 24 h after each time point. The vaccine induced an antibody response in 9/11 volunteers, and there was an association between protein levels of GM-CSF, CXCL10 in serum monocytes and the antibody titer at the final visit.

Many of the genes upregulated were in cytokine-related pathways. In a similar study measuring transcriptomic changes after human immunization with saRNA, changes in many of the same genes were observed,^12^ and we also observed similar gene upregulation in a muscle cell line.^7^ Both studies used LNP-encapsulated saRNA—the formulation impacts vaccine sensing by the innate immune response and influences the downstream adaptive immune response.^3^ There were also similarities with acute gene expression profiles in response to non-replicating mRNA vaccines,^11^^,^^13^^,^^14^^,^^15^ suggesting that this is a shared response to LNP-RNA and not specific to saRNA. There were 11 upregulated genes in common across these four studies, including ours: *SERPING1*, *MX1*, *IFIT2*, *IFI35*, *IFIH1*, *SOCS1*, *GBP1*, *PML*, *STAT2*, *STAT1*, and *GBP5*, nearly all of which are linked to the type I IFN response. These data indicate that there is potentially a core signature of transcriptomic responses to RNA-based vaccines in humans, agnostic of the RNA modality used.

A key question is how genes upregulated 24 h after immunization influence the downstream adaptive response, specifically vaccine efficacy and effectiveness. Because m1Ψ dampens type I IFN responses *in vitro* and in pre-clinical models, and vaccines containing modified nucleotides had greater efficacy than those that did not,^16^ the widely held assumption is that type I IFN responses are deleterious for RNA vaccines. However, some level of inflammation may also be required to activate the immune system to recognize the encoded antigen.^8^ There is a critical role for RNA vaccine-induced inflammation in recruiting and activating antigen-presenting cells.^3^ With the caveat of a small study size, the cytokine response in the blood and the associated increase in monocytes correlated with the antibody titer. This reflects what we have previously seen in mouse models, with a correlation between blood CXCL10 24 h after immunization and antibody titer.^3^ Whether CXCL10 is functionally important in the cascade leading to an antibody response or acts as a biomarker needs further dissection. CXCL10 is a monocyte chemoattractant, and we observed a significant correlation between monocyte percentage at V2a and antibody responses, suggesting that these cells play an important role—potentially through shuttling antigen to lymph nodes or presenting antigen to T cells. Monocyte signatures have been linked to antibody responses to mRNA vaccination^11^; interestingly, this study also observed an increase in early NK cells, as we did. A similar correlation was observed with CCL2^3^^,^^12^; CCL2 expression closely correlated with monocyte recruitment in our study. Further exploration is required, but^11^ the overlap with mouse models opens opportunities for mechanistic dissection.

Other studies have observed a change in the magnitude of the transcriptomic response after booster vaccination with RNA.^11^^,^^15^^,^^17^ We saw a similar effect in this study, with genes in the B8 module identified by Arunachalam et al. showing significantly greater expression at the boost immunization.^11^ The reason for this boosted response is as yet unclear. Several different mechanisms have been proposed. One study suggests that there are epigenetic changes in monocytes, particularly in ISREs (IFN-stimulated response elements), though this was performed in a very small number of volunteers (*n* = 4).^17^ A study using an adjuvanted protein vaccine against influenza virus has also proposed changes in chromatin accessibility in myeloid cells.^18^ Alternatively, mouse studies suggest a role for IFN-γ production by CD4 and CD8 T cells, which, when blocked, reduces the increases in gene expression.^10^ A role for primed mucosal-associated invariant T (MAIT) cells has also been proposed, although this was in response to an adenovirus vaccine.^19^

We compared responses based on whether there had been any moderate adverse events, either systemic or local. The numbers in each group were very small, and the grouping around adverse effects has many limitations—not least the subjective nature of adverse effect scoring. These reflect wider limitations of the study; it was a very small study, nested within a larger study. However, it does give a potential window into the causes of adverse events following vaccination and whether there are aspects that can be modified without altering the downstream adaptive immune response. There appeared to be some signatures associated with reactogenicity, with the DEGs unique to these individuals mapping to GO terms associated with the immune response.

In the current study, we have described the innate response to an saRNA vaccine in antigen-naive individuals and shown that vaccination is linked to increased cytokine production and cell recruitment. One of the limitations of the study is that it was not sufficiently powered to investigate the links between transcriptomic responses to vaccination and downstream adaptive immune responses. This was because the study was planned to recruit only antigen-naive individuals, and therefore the rollout of licensed COVID-19 vaccines ultimately limited the number of volunteers in the study. Future studies using a systems vaccinology approach are required to address questions about the mechanisms by which saRNA induces protection against infection.

## Materials and methods

### Study design and participants

To assess a second-generation saRNA vaccine (LNP-nCoVsaRNA-02), 11 healthy participants aged 18−45 years were recruited through local advertisements. Participants were randomized into groups to receive two doses of either a 1 or a 5 μg dose of LNP-nCoVsaRNA-02 via intramuscular vaccination into the deltoid, with intervals of 4 to 8 weeks (Table S1). Participants and laboratory staff were blinded to allocation.

Participants with no history of COVID-19 or vaccination were eligible to take part. All participants underwent a screening visit, during which a full medical history and examination were performed, in addition to blood and urine tests. Participant sera were screened for antibodies against SARS-CoV-2 using the Abbott Architect nucleocapsid IgG assay (N IgG), for the presence of blood-borne viruses using a fourth-generation HIV test, and for IgG against Hepatitis C. Those with reactive responses in any of these tests were ineligible for the study. Full details of the eligibility criteria are described in Doc S1.

Written informed consent was obtained from all participants, and the trial was conducted in accordance with the principles of the Declaration of Helsinki and Good Clinical Practice. Participants were offered reimbursement for their time, inconvenience, and travel expenses of £50 per visit, paid as a lump sum at the end of the participation. This study was approved in the UK by the Medicines and Healthcare products Regulatory Agency and the North East - York Research Ethics Committee (ref. 20/SC/0145) (ISRCTN17072692, EudraCT 2020- 001646-20).

### Vaccine

LNP-nCoVsaRNA-02 is a saRNA vaccine, 11,507 nucleotides in length, encapsulated within LNPs. It encodes two major components: the non-structural replicase proteins from the VEEV epizootic Trinidad Donkey strain, and the spike (S) glycoprotein of SARS-CoV-2 (GenBank accession number: QHD43416.1), stabilized in the prefusion conformation by the insertion of two proline substitutions, K986 and V987.9. It also includes ORF4a from MERS-CoV.^20^ The saRNA is generated from a DNA template by *in vitro* transcription with co-transcriptional capping (m7G(5)ppp(5) (2OMeA)pU; Tri-Link). An additional sequence of 109 amino acids, encoding the open reading frame (ORF) 4a protein from MERS, was incorporated within this vaccine. The RNA is formulated with lipids—ionizable lipid (proprietary to Acuitas), phosphatidylcholine, cholesterol, and PEG-lipid—to obtain the LNP-nCoVsaRNA drug product.

The vaccine was formulated as a suspension for injection in multi-dose vials stored at −70°C. On the day of injection, it was thawed and diluted in phosphate-buffered saline (PBS) to give a final volume of 0.5 mL for injection for dose levels from 1.0 to 5.0 μg.

### Participant samples

PBMCs were isolated using density gradient separation from heparinized whole blood. Cells were processed within 4 h of collection and stored at 1 × 10^7^ cells/mL in freezing media (10% dimethyl sulfoxide, 90% fetal bovine serum; Sigma-Aldrich) at −80°C before storage in ultra-low −150°C conditions prior to analysis by flow cytometry.

Whole blood was collected into RNA Paxgene tubes (BD 762165). The tubes were stored upright and frozen at −80°C prior to transcriptomic analysis.

### MSD method

Baseline and 24-h post vaccination COVAC1 plasma samples at visits 2, 2a (prime), 5, 5a (boost), and 7 were run on a MSD platform to assess cytokine and chemokine levels.

Thirteen markers of interest (CXCL5, CCL24, CXCL1, GM-CSF, IFN-β, IL-1α, IL-1β, CXCL10, CCL2, CCL7, CCL22, CCL3, and CCL4) were analyzed following the U-PLEX Biomarker kit protocol, described briefly: 96 well MSD plates were coated with the biotinylated linker complex and kept at 2°C–8°C for up to 7 days until the day of assay. Diluted calibrator reference, at a 5-fold serial dilution, and plasma samples were added to the plates for 1 h at room temperature with shaking. Plates were washed, and SULPHO-TAG detection antibody was added for 1 h before washing. MSD GOLD read buffer was added immediately before reading the plate on an MESO QuickPlex SQ 120MM. Data were analyzed using MSD DISCOVERY Workbench software.

### RNA-seq

Total RNA was extracted using the Qiagen PAXgene Blood RNA Kit, following the manufacturer’s instructions (Qiagen, Hilden, Germany). RNA-seq library preparation was performed using the NEBNext Ultra II Directional RNA Library Prep Kit for Illumina, following manufacturer’s instructions (NEB, Ipswich, MA, USA).

The sequencing libraries were multiplexed and loaded onto the flowcell of the Illumina NovaSeq instrument, according to the manufacturer’s instructions. The samples were sequenced using a 2 × 150 pair-end (PE) configuration, v1.5. Image analysis and base calling were conducted by the NovaSeq Control Software v1.7 on the NovaSeq instrument. Raw sequence data (.bcl files) generated from the Illumina NovaSeq were converted into FASTQ files and de-multiplexed using the Illumina bcl2fastq program, version 2.20. One mismatch was allowed for index sequence identification.

Raw read data were mapped to the human genome (GENCODE v39) using STAR v2.7.10a. STAR was run with the option “--quantMode GeneCounts », which automatically converts the alignment information into gene-level counts. From the manual: “With --quantMode GeneCounts option, STAR will count the number of reads per gene while mapping. A read is counted if it overlaps (1 nt or more) one and only one gene. Both ends of the paired-end read are checked for overlaps. The counts coincide with those produced by htseq-count with default parameters.”

Genes that did not have more than 0.389 counts per million in at least 10 samples were filtered out. Differential expression was estimated through the limma-voom workflow.

TMOD analysis was performed using the tmod package in R.^21^ The number of significant genes per module was calculated using the function tmodDecideTests and visualized using the ggPanelplot function from the cowplot package (version 1.1.3).

### SARS-CoV-2-spike-specific IgG ELISA

Sera were heat-inactivated for 30 min at 56°C prior to storage at −20°C, before assessment in immunological assays. Binding antibody concentrations induced by the vaccine in participant sera were assessed using an anti-S IgG ELISA in 96-well plates coated with the stabilized SARS-CoV-2 spike protein in the pre-fusion conformation. Background optical density (OD) 450 nm readings from uncoated wells (blank wells) were subtracted from readings in test wells. Vaccine-induced seroconversion to anti-S IgG was considered to have occurred in those with an OD above 0.2 nm in the ELISA. This threshold was set during optimization of the ELISA using standards from the National Institute for Biological Standards and Control (NIBSC), convalescent sera from individuals who had recovered from SARS-CoV-2 infection, and sera taken prior to December 2019. Baseline convalescent sera were available from 32 individuals enrolled in the wider COVAC1 trial, with either a history of mild or moderate COVID, or previously unknown, asymptomatic SARS-CoV-2 infection. A positive control of pooled plasma samples from NIBSC was included in each assay plate. When it became available (December 2020), the first WHO international standard anti-SARS-CoV-2 immunoglobulin was added at a concentration of 2 BAU/mL as an additional control. It was determined that 2 BAU/mL was equivalent to approximately 20,000 ng/mL.

### SARS-CoV-2 neutralization assay

Briefly for the PSV assay, pseudotyped SARS-CoV-2 lentiviruses were produced in HEK293T/17 cells using a SARS-CoV-2 spike plasmid, an HIV-1 gag-pol plasmid, and a firefly luciferase reporter. Participant sera were serially diluted and incubated with PSV viral supernatant for 1 h. HEK-ACE2 cells were then co-incubated with the sera and PSV for up to 96 h before measurement of luciferase activity using the Steady-Glo Luciferase assay system (Promega, Madison, WI). IC50 neutralization titers were calculated as the dilution at which relative luminescence was reduced by 50% compared with the control. For the PSV assay, the first WHO International Standard for anti-SARS-CoV-2 immunoglobulin was included as a positive control, which was determined to have an IC50 neutralization titer of approximately 1:3,000.

### IFN-γ ELISpot

IFN- γ ELISpot assays were performed using frozen, isolated PBMCs stimulated with peptide pools matched to the vaccine at sampling weeks, 0, 4, and 6. In brief, frozen PBMCs were rested overnight at a concentration of 2.5 × 106 PBMCs/mL and re-suspended to a final concentration of 4 × 106 viable PBMCs/mL (4 × 105 PBMCs/mL for positive control phytohemagglutinin [PHA] wells). Pre-coated IFN- γ ELISpot 96-well plates (Mabtech) were washed with sterile PBS and blocked with R10 media before the addition of 50 μL of cells per well in triplicate, with 50 μL media only, stimulation media containing vaccine-specific peptide pools Env 1, 2, and 3 (15-mers overlapping by 11, covering the entire sequence of SARS-CoV-2 spike matched to the vaccine insert), and two positive controls, PHA (Sigma-Aldrich) and CEFX Ultra SuperStim Pool (JPT, Berlin, Germany), at a final concentration of 2.5 μg/mL. Plates were incubated for 16–24 h at 37°C, 5% CO2. Plates were then washed and incubated for 2 h at room temperature with 1 μg/mL mouse-anti-human IFN-γ (Mabtech). The signal was amplified with a 1-h incubation with streptavidin-ALP solution (Mabtech), then developed with substrate, 5-bromo-4-chloro-3-indolyphosphate and nitro-blue tetrazolium (BCIP/NBT plus; Mabtech) for 5–7 min. The reaction was stopped by washing with tap water and allowed to dry overnight in the dark. Plates were read with an automated AID iSPOT ELISpot plate reader (Autoimmun Diagnostika GmbH). The number of spot forming units (SFU)/106 PBMCs was calculated as the mean count after subtracting the background count. Pass/fail criteria for the assay were dependent on the mean of the negative wells being <100 SFU/106 PBMCs, and the PHA-positive response being >500 SFU/106 PBMCs.

### Cell phenotyping by spectral flow cytometry

A 35-color panel was developed to evaluate PBMC populations at visits 2, 2a, 5, and 5a (Table S2). Briefly, frozen PBMCs were thawed and rested for 2 h at 37°C, 5% CO2 before staining. Cells were sequentially incubated with anti-CCR7, Brilliant Stain Buffer Plus, anti-CX3CR1 and -CD64, True-Stain Monocyte Blocker, anti-IgG, Human TruStain FcX, anti-CCR5, other anti-chemokines (CXCR3, CCR2, and CXCR5), and a mix of all other antibodies. The stained cells were fixed with 1X BD CellFix (BD Biosciences) before acquisition on the Aurora spectral cytometer (Cytek Biosciences) and analyzed using FlowJo v10.10.0 (BD Biosciences) and the OMIQ platform (https://www.omiq.ai). We analyzed the abundance of different blood cell subpopulation as indicated in the gating strategy (Figure S5).

### Associations with reactogenicity

Individual participants were grouped by whether they had adverse effects of grade 2 or greater (G2) or not (noG2) after the prime, based on a self-reported survey. A principal-component analysis (PCA) plot was first generated using the PCAtools package (version 3.19), and the loading plot was generated using the function biplot. Differential GO pathways responsible for each principal component was analyzed using PGSEA to identify gene sets and corrected for multiple testing. This was visualized using smcPlot. A differential gene analysis was carried out using DeSeq2^22^ to compare V2a to V2 in the G2 and noG2 populations. This produced a list of genes with associated *p* values, adjusted *p* values, and log2 fold changes, with positive log fold change values indicating increased gene expression and negative values indicating decreased gene expression. False discovery rate (FDR) was calculated by applying the weighted Benjamin–Hochberg method for multiple hypothesis testing. A gene was considered differentially expressed if the absolute fold change was above 0 with adjusted *p* value < 0.05. GO analysis was carried out using ClusterProfiler^23^ to assess the enrichment of GO pathways in each gene list. Network analysis of the GO terms was analyzed using the emapplot function in the ClusterProfiler package.

### Comparative transcriptomics

Raw readings from Ong^12^ (comparing day 1 and 2 post-vaccination), Lee^14^ (comparing day 0 and 1), and Arunachalam^11^ (comparing baseline and Day 1.2) were analyzed. DEseq2 was used for differential analysis, and a gene was considered differentially expressed if the absolute fold change was above 0 with an adjusted *p* value < 0.05. Volcano plots were generated using VolcaNoseR^24^ for each comparison.

## Data and code availability

Data is available https://doi.org/10.82186/hg8d9-tc025: Differentially expressed genes (Table S3); Total gene counts (Table S4).

## Acknowledgments

This study was co-funded by grants and gifts from the 10.13039/501100000265Medical Research Council UKRI (MC_PC_19076), the 10.13039/100000002National Institute Health Research/Vaccine Task Force, Partners of Citadel and Citadel Securities, Sir Joseph Hotung Charitable Settlement, 10.13039/501100023262Jon Moulton Charity Trust, Pierre Andurand, and Restore the Earth. R.J.S. received a gift from the James B. Pendleton Charitable Trust for equipment used in this study. An 10.13039/100012411NIHR BRC award to Imperial supported J.S.T. and R.J.S. Z.W. was funded in part by the Department of Infectious Disease, 10.13039/501100000761Imperial College London, as part of the 10.13039/100000152Molecular and Cellular Basis of Infection program, and in part by a 10.13039/100010269Wellcome Trust studentship. The authors thank Lesley Rawlinson for lab management, Selma Bennacer for support, and Sheena McCormack and Henry Bern (MRC Clinical Trials Unit at UCL, London, UK) for help with access to data.

## Author contributions

T.E. (investigation), Z.W. (formal analysis), O.B. (investigation, supervision, formal analysis, writing – review & editing), A.E. (investigation), S.D. (investigation), L.R.M. (investigation), S.d.B. (formal analysis), K.A. (formal analysis), J.N. (formal analysis), M.W. (investigation), K.P. (investigation), H.M.C. (supervision), B.C. (supervision, conceptualization, writing – review & editing), R.J.S. (supervision, funding acquisition, conceptualization), J.S.T. (writing – first draft, supervision).

## Declaration of interests

R.J.S. is a co-inventor on a patent application covering this SARS-CoV-2 saRNA vaccine.

## Supplemental information

Document S1. Figures S1–S5 and Tables S1 and S2

Table S3. Differential expressed genes

Table S4. Total gene counts/ raw data by volunteer

Table S5. Differential genes between G2 and no G2

Document S2. Article plus supplemental information
