# Supplementary material for: Systems vaccinology analysis of saRNA immunization identifies an acute innate immune signature correlated with adaptive immunity
Source: Mol Ther Adv. 2026 Feb 20;34(1):201706. doi: 10.1016/j.omta.2026.201706 (PMC13148895; doi:10.1016/j.omta.2026.201706)
Supplement: Document S1. Figures S1–S5 and Tables S1 and S2 [file mmc1.pdf]

OMTA, Volume *34*

Supplemental information

Systems vaccinology analysis of saRNA immunization identifies an acute innate immune signature correlated with adaptive immunity

Tamara Elliott, Ziyin Wang, Olivia Bonduelle, Abbey Evans, Suzanne Day, Leon R. McFarlane, Simon de Bernard, Karine Alves, Julien Nourikyan, Michele Wokam, Katrina Pollock, Hannah M. Cheeseman, Behazine Combadiere, Robin J. Shattock, and John S. Tregoning

Supplemental Material

Table S1 Volunteers by immunisation schedule and adverse effects.

**Volunteer**	**Dose and schedule**	**Assigned to dose group**	**Adverse effects (Grade 2 or no Grade 2)**
Vol1	1.0µg at week 0 and 1.0µg at week 4	1µg	No G2
Vol2	5.0µg at week 0 and 5.0µg at week 4	5µg	No G2
Vol3	5.0µg at week 0 and 5.0µg at week 4	5µg	No G2
Vol4	1.0µg at week 0 and 1.0µg at week 4	1µg	No G2
Vol5	1.0µg at week 0 and 1.0µg at week 4	1µg	No G2
Vol6	5.0µg at week 0 and 5.0µg at week 4	5µg	No G2
Vol7	5.0µg at week 0 and 5.0µg at week 8	5µg	G2
Vol8	5.0µg at week 0 and 5.0µg at week 4	5µg	No G2
Vol9	5.0µg at week 0 and 5.0µg at week 8	5µg	G2
Vol10	5.0µg at week 0 and 5.0µg at week 4	5µg	G2
Vol11	1.0µg at week 0 and 1.0µg at week 8	1µg	No G2

Table S2: Flow panel used

Reagent	Clone	Source	Catalog number
Mouse anti-human CD8 BUV395	RPA-T8	BD Biosciences	563795
LIVE/DEAD™ Fixable Blue Dead Cell		Molecular Probe	L34962
Mouse anti-human CD16 BUV496	3G8	BD Biosciences	612944
Mouse anti-human CD45RA BUV563	HI100	BD Biosciences	612926
Mouse anti-human CD20 BUV615	2H7	BD Biosciences	751256
Mouse anti-human CCR7 BUV661	2-L1-A	BD Biosciences	749824
Rat anti-human CX3CR1 BUV737	2A9-1	BD Biosciences	749355
Mouse anti-human CD4 BUV805	SK3	BD Biosciences	612887
Mouse anti-human CD279 (PD1) BV421	EH12.2H7	BioLegend	329920
Mouse anti-human CD123 Super Bright 436	6H6	eBioscience	62-1239-42
Mouse anti-human IgG V450	G18-145	BD Biosciences	561299
Mouse anti-human CD14 PerCP-Cy5.5	MΦP9	BD Biosciences	562692
Mouse anti-human CD11b BV510	ICRF44	BD Biosciences	563088
Mouse anti-human HLA-DR BV570	L243	BioLegend	307638
Mouse anti-human CD56 BV605	NCAM16.2	BD Biosciences	562780
Mouse anti-human CXCR3 BV650	B025H7	BioLegend	353730
Mouse anti-human CXCR5 BV711	J252D4	BioLegend	356934
Mouse anti-human CCR2 BV750	LS132.1D9	BD Biosciences	747856
Mouse anti-human IgM BV785	MHM-88	BioLegend	314544
Mouse anti-human CD141 BB515	1A4	BD Biosciences	565084
Mouse anti-human CD3 SparkBlue550	SK7	BioLegend	344852
Mouse anti-human CD45 PerCP	2D1	BioLegend	368506
Mouse anti-human IgD BV480	IA6-2	BD Biosciences	566138
Mouse anti-human CD11c PerCP-Vio700	MJ4-27G12	Miltenyi Biotec	130-113-582
Mouse anti-human CD10 PE	HI10a	BioLegend	312204
Mouse anti-human CCR5 PE/Dazzle 594	J418F1	BioLegend	359126
Mouse anti-human CD28 PE-Cy5	CD28.2	BD Biosciences	555730
Mouse anti-human CD25 PE-Cy5.5	B1.49.9	Beckman Coulter	B92458
Mouse anti-human CD27 PE-Cy7	M-T271	BD Biosciences	560609
Mouse anti-human CD161 APC	HP-3G10	BioLegend	339912
Mouse anti-human CD1c AF647	L161	BioLegend	331510
Mouse anti-human CD19 Spark NIR 685	HIB19	BioLegend	302270
Mouse anti-human CD127 APC-R700	HIL-7R-M21	BD Biosciences	565185
Mouse anti-human CD64 APC-H7	10.1	BD Biosciences	561190
Mouse anti-human CD38 APC-Fire810	HIT2	BioLegend	303550
Brilliant stain buffer Plus		BD Biosciences	566385
True-Stain Monocyte Blocker		BioLegend	426103
Human TruStain FcX		BioLegend	422302
BD CellFix 10X		BD Biosciences	340181

Additional Supplemental Tables
Table S3 – Differential Expressed Genes
Table S4 – Total gene counts/ raw data by volunteer.
Table S5 – Differential genes between G2 and no G2

A

	V2a-V2 up	V2a-V2 down	V5a-V5 up	V5a-V5 down
**1μg**	386	16	997	170
**5μg**	4607	4213	4635	4551

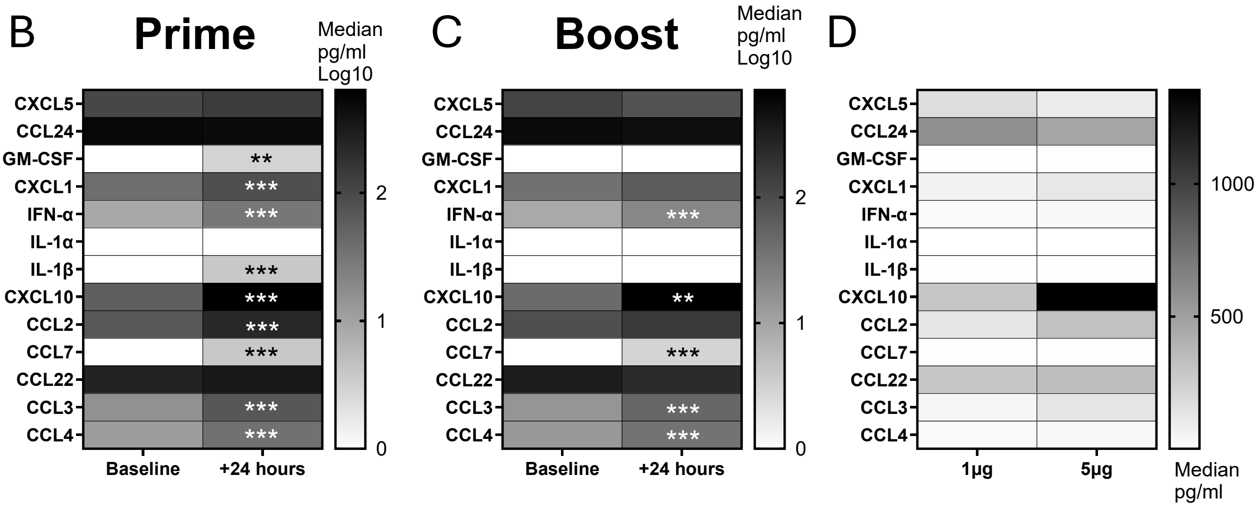

Figure S1. Increased level of cytokines in blood after immunisation. A) Comparison of DEG by dose of RNA received. B) MSD data for V2a (prime) separated by dose of RNA received. C) MSD cytokine data from plasma for V5a (boost) D) MSD cytokine data from plasma after prime by dose. *** p<0.001, **** p<0.0001.

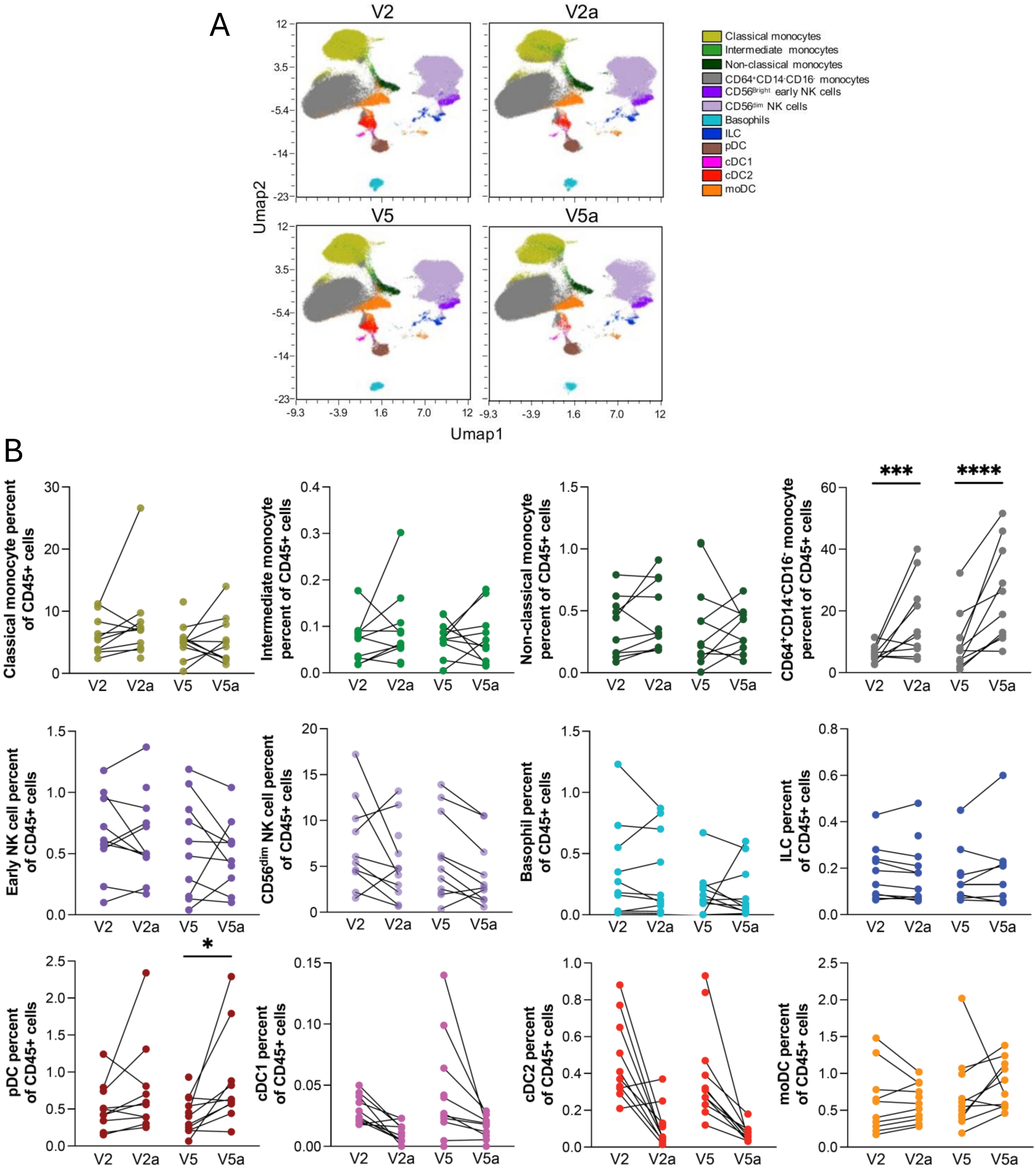

Figure S2. Increased level of innate cells in blood after immunisation. A) UMAP (Uniform Manifold Approximation and projection) was performed on 15,000 randomly chosen PBMC (without B and T cells) from 11 subjects at V2 (upper left), V2a (upper right), V5 (bottom left) and V5a (bottom right). 12 innate subpopulations were analysed. B) Plots show percent of blood cell populations of each subject at V2, V2a, V5 and V5a; colours match UMAP. *** p<0.001, **** p<0.0001

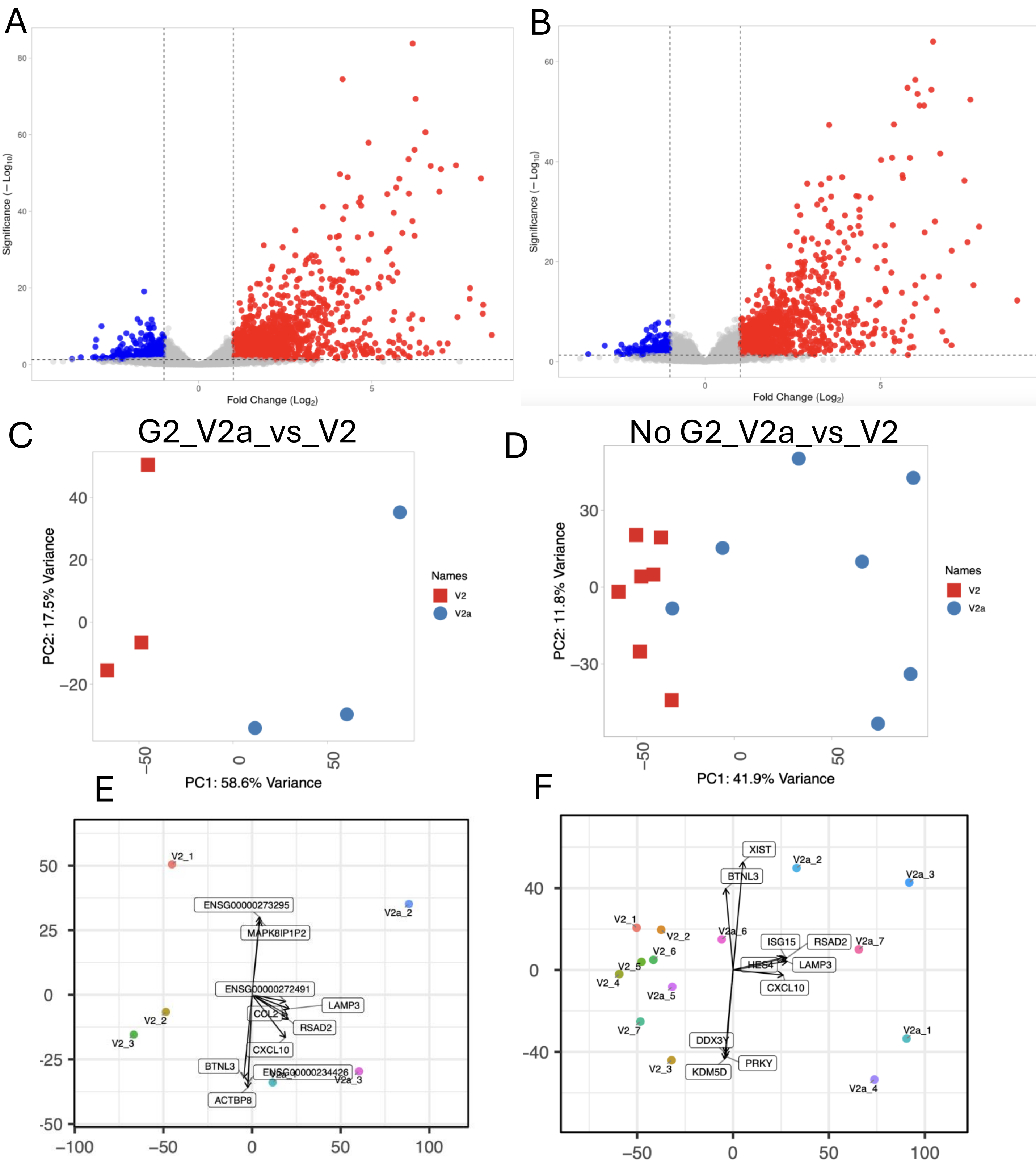

Figure S3. Differential response between baseline and 24 hours in individuals with and without adverse effects. Participants were clustered by whether they experienced a grade 2 adverse effect (G2) or not (No G2). Volcano plot comparing DEG between V2 and V2a for G2 (A) or no G2 (B). PCA of responses (C, D); loading plots of PCA (E, F).

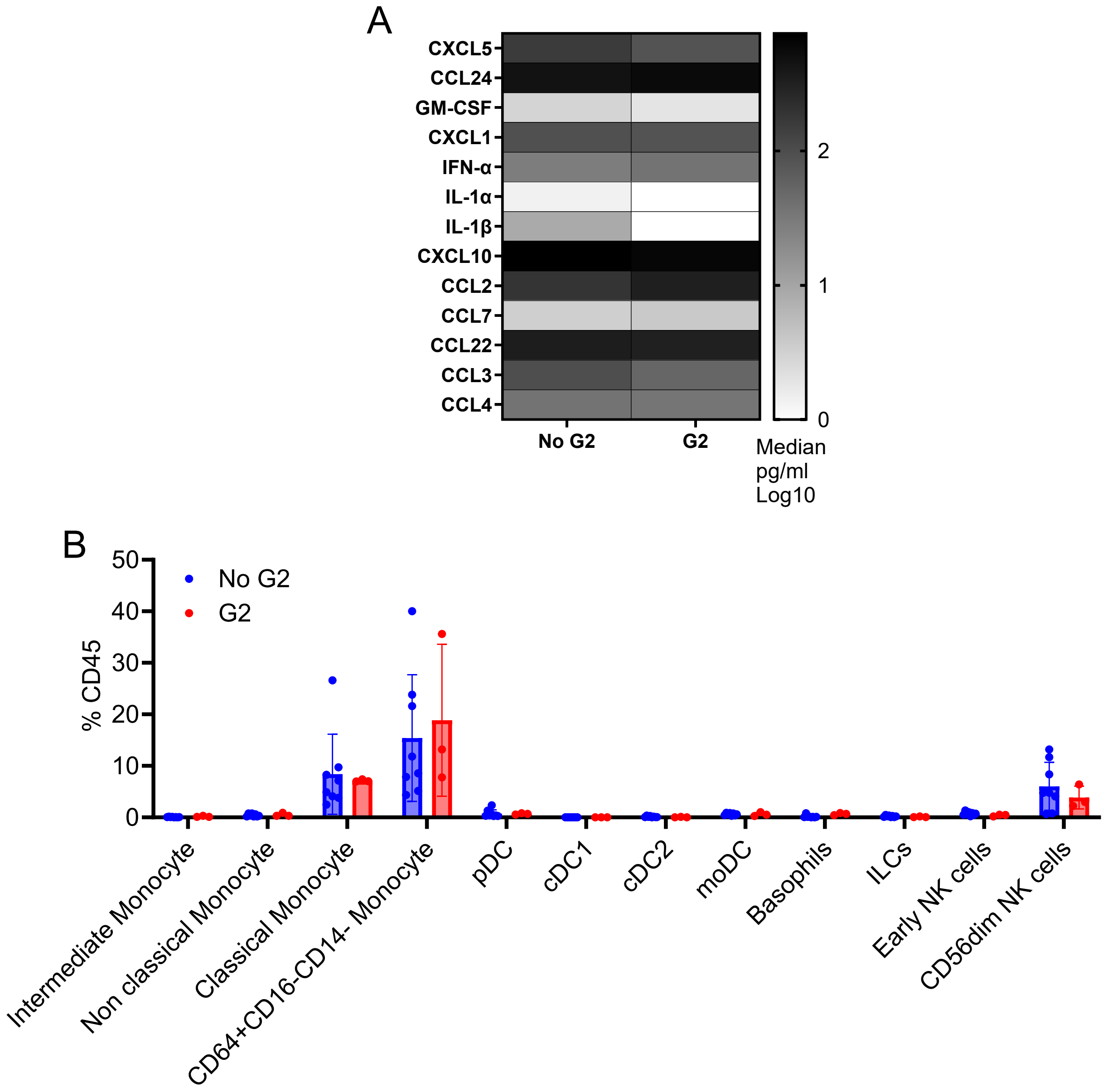

Fig S4. Comparison of innate response by adverse effect grouping. A) MSD cytokine data from plasma at V2a separated by adverse effects. B) cell typing by flow cytometry grouped by adverse effects.

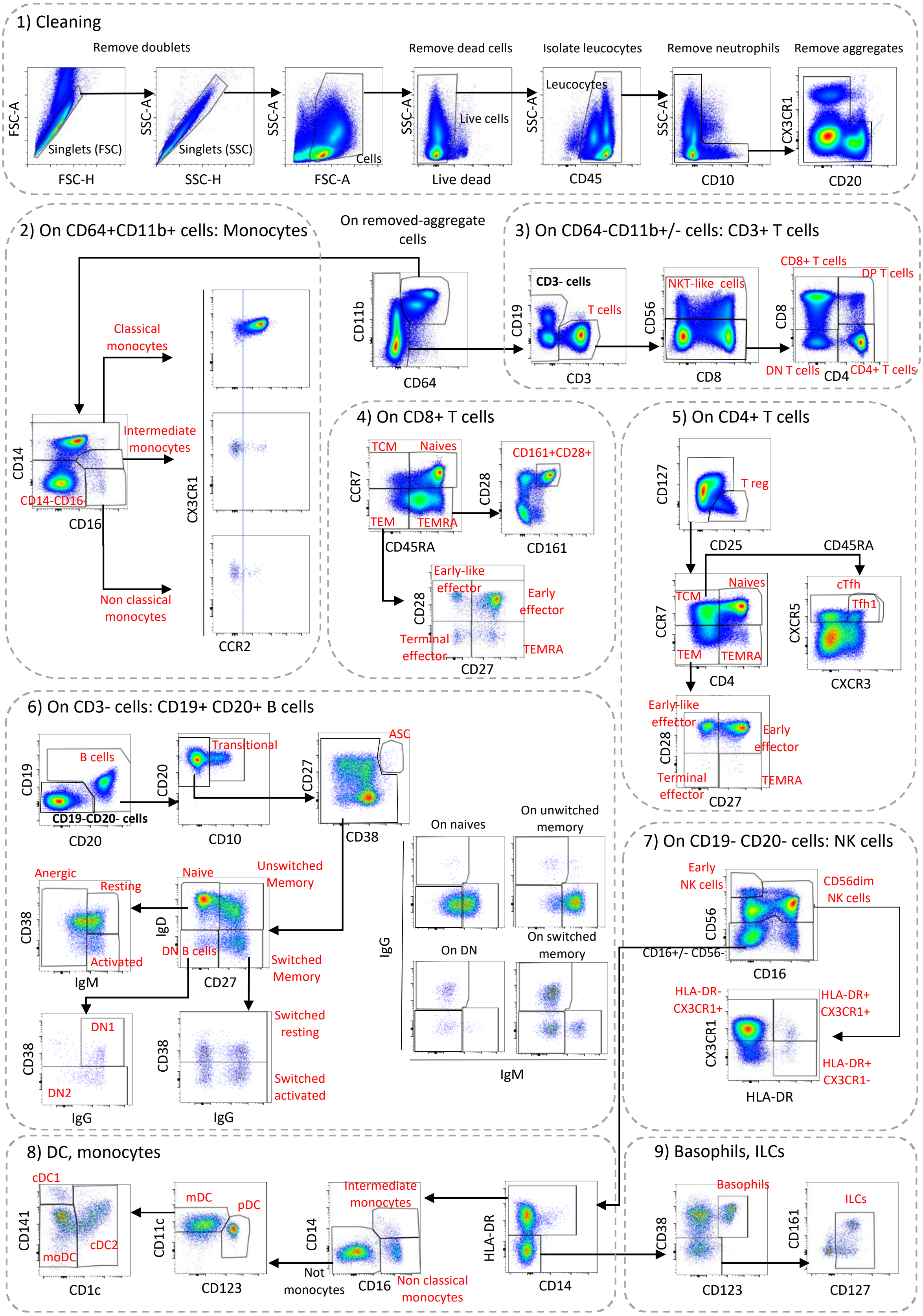

Fig S5. Gating strategy for flow analysis.
